# Patient’s early satisfaction with care: a predictor of health-related quality of life change among outpatients with substance dependence

**DOI:** 10.1186/s12955-019-1267-x

**Published:** 2020-01-07

**Authors:** Ophélie Müller, Cédric Baumann, Paolo Di Patrizio, Sarah Viennet, Guillaume Vlamynck, Laura Collet, Isabelle Clerc-Urmès, Raymund Schwan, Stéphanie Bourion-Bédès

**Affiliations:** 1 CSAPA (Health Care Center of Accompaniment and Prevention in Addictology), University Hospital of Nancy, 54000 Nancy, France; 2 Unit of Methodology, Data Management and Statistics, University Hospital of Nancy, 54500 Vandoeuvre-lès-Nancy, France; 3 EA4360 APEMAC (Health adjustment, measurement and assessment, interdisciplinary approaches) MICS team, University of Lorraine, 54500 Vandoeuvre-lès-Nancy, France; 4 Service médico-psychologique régional, 1, Rue Seulhotte B.P, 15082 57073 Metz, France

**Keywords:** Health-related quality of life, Determinants, Satisfaction, Outpatient, Substance dependence

## Abstract

**Background:**

Although research on health-related quality of life (HRQoL) has increased in the addiction field, few studies have focused on the determinants of HRQoL changes. This study aimed to describe dependent patients’ HRQoL changes at a 3-month follow-up and to assess whether satisfaction with care can predict those changes among outpatients starting care for alcohol or opioid dependence.

**Methods:**

HRQoL was measured with the SF-12 at baseline and 3 months later in a prospective cohort of dependent outpatients. Satisfaction was assessed with the EQS-C early after inclusion. Data on sociodemographics, clinical characteristics and patients’ levels of anxiety and depression were also collected. A multivariable analysis was performed to identify factors associated with HRQoL changes in both the physical and mental component summary scores (PCS and MCS, respectively).

**Results:**

Of the 172 patients included at baseline, a total of 136 patients assessed their satisfaction with care. The mean PCS and MCS scores were initially low, and HRQoL improvement was significant after 3 months for both the PCS and MCS. Never having been married (β = 5.5; *p* = 0.001) and a lower baseline PCS score (β = − 0.6; *p* < 0.0001) were associated with significant PCS improvement, whereas being legally compelled to undergo drug treatment (β = − 5.9; *p* = 0.02) was associated with less PCS change. Higher early satisfaction with care (β = 0.1; *p* = 0.02) and a lower baseline MCS score (β = − 0.7*; p* < 0.0001) were associated with significant MCS improvement.

**Conclusion:**

The study supported the hypothesis that greater satisfaction with care may predict HRQoL improvement among dependent outpatients. Further studies are needed to understand the factors that affect patients’ early satisfaction to identify areas of improvement and thus improve HRQoL.

## Introduction

Given the chronic, relapsing nature of substance use disorder (SUD) [1, 2] and the negative consequences in various life domains affected by drug use [3], there has been expanding interest in measuring patient-reported outcomes in people with SUDs in recent years. Numerous studies have included health-related quality of life (HRQoL) and quality of life (QoL) assessment as an important clinical and research tool, as an outcome for assessing the health of patients with SUDs and for evaluating drug programs [4–6]. Although QoL and HRQoL are different (QoL is an all-inclusive concept incorporating all factors that impact an individual’s life, while HRQoL includes only those factors related to an individual’s health), they both aim to capture a patient’s subjective perception and assessment of his or her health and well-being [7, 8]. Currently, there is evidence that QoL will improve as a function of treatment and recovery in patients with SUDs [9]. Moreover, it has been suggested that QoL should be assessed and reported regularly by clinicians from the beginning of addiction treatment to support evaluations of the recovery progress and decision making with regard to continuing care [10].

Among individuals with SUDs, HRQoL is generally poorer than that of the general population and as low as that of individuals with other chronic diseases or serious psychiatric disorders [11–13]. Several sociodemographic and clinical variables have been studied as predictors of baseline QoL among patients suffering from SUD [14, 15]. The findings are somewhat inconsistent and difficult to interpret because of differences in methodologies, instruments and populations [16]. Overall, male gender, younger age, higher education and being employed are consistently associated with better QoL scores [17, 18]. Inversely, suffering from physical and mental comorbidities altered QoL [19, 20]. Regarding the main SUD-specific characteristics, the severity of dependence is constantly associated with poorer functioning in nearly all QoL domains [21], whereas the duration of addiction, drinking patterns and prior treatments are not [22]. It has also been noted that opiate dependence impaired QoL more than alcohol dependence did [23]. Only a few studies have focused on factors associated with HRQoL improvement among the SUD population. Although the literature has shown that specialized SUD treatment enhanced QoL for dependent patients [9], divergent findings have been reported regarding the predictors of substantial improvements in HRQoL [24]. Indeed, some studies provided evidence that QoL improved with abstinence [25], whereas others showed that there was no correlation between a reduction in substance use and HRQoL [26]. In addition, a published study found that sociodemographic and clinical factors, such as marital status, income and somatic or psychological comorbidities, explained differences in QoL changes between alcohol-dependent twins and their abstinent cotwins [27]. Similarly, the relationship between patients’ satisfaction with care and QoL changes has not been clearly established among populations with psychiatric disorders [28]. Patient satisfaction can be defined as an individual’s cognitive evaluation of and emotional reaction to his or her health care experience [29, 30]. Particularly important to the provision of quality addiction services, patient satisfaction has been found to predict better treatment outcomes, including better physical and mental health [31] and psychological improvements [32]. Although many studies have explored HRQoL and SUDs, few have analyzed changes in these scores and determinants of their improvement, and no previously published study has focused on patients’ early satisfaction as a factor related to changes in HRQoL.

Thus, this study aimed to a) examine patients’ HRQoL changes at a 3-month follow-up and b) identify whether early satisfaction with care predicted a change in HRQoL among outpatients who are starting care for alcohol or opioid dependence.

## Methods

### Participants and setting

This study was based on a longitudinal analysis of data from the SUBstance Users Satisfaction and Quality Of Life (SUBUSQOL) cohort. This is a prospective cohort of outpatients aged over 18 years who began care at French specialized addiction treatment centers and met the Diagnostic and Statistical Manual of Mental Disorders, fourth edition (DSM-IV) [33] criteria for alcohol dependence or opioid dependence (ClinicalTrials.gov ID: NCT02894476). The participants were recruited by clinicians who were certified in addiction pathologies. The treatment wards had multidisciplinary staff, including psychiatrists, psychologists, social workers and specialized nurses. Treatment included individual motivation enhancement, supportive therapy, pharmacotherapy and assessments of somatic and mental health.

### Data collection

Upon entry into the SUBUSQOL study, sociodemographic and clinical data were collected at the time of inclusion (T0) and 3 months after inclusion (T2) through medical interviews and clinical testing. HRQoL and anxiety-depression were assessed with self-reported questionnaires at T0 and T2. Satisfaction was assessed with a self-administered questionnaire to be completed at home 15 days after the first visit (T1). Outpatients who returned the satisfaction with care questionnaire comprised the cohort for the present set of analyses.

#### Health-related quality of life

Health-related quality of life was assessed with the Short-Form 12 questionnaire (SF-12), which is a generic 12-item instrument based on the earlier SF-36 [34]. The French version has yielded valid and reliable clinical assessments of self-reported health status among substance users [35, 36]. The SF-12 covers eight domains: physical functioning, role-physical (that is, role limitations due to physical problems), bodily pain, general health, vitality, social functioning, role-emotional (that is, role limitations due to emotional problems) and mental health. Information from all 12 items is used to calculate a physical health component summary (PCS) and a mental health component summary (MCS). All scores were transformed to a standardized 0–100 score, with higher scores indicating better HRQoL.

#### Outpatient satisfaction

Satisfaction was assessed with the quality of care satisfaction in outpatient consultation questionnaire (EQS-C), for which validity and reliability have been previously established [37]. The EQS-C self-report questionnaire includes 27 items assessing 4 dimensions that explore different aspects of care and satisfaction with staff and treatment: contact/appointments (6 items), reception facilities (5 items), waiting time (3 items) and consultation with the doctor (13 items). Each item is scored from 0 to 4, with 4 indicating the greatest level of satisfaction. A “does not apply” category is provided for 13 items related to situations that are not universally relevant. Nonresponses and selection of the “does not apply” category were considered missing data. Scores were computed when at least half of the items in a dimension were completed. The score for each dimension was calculated by summing the items. All scores were transformed to a standardized 0–100 score, with higher scores indicating greater satisfaction. The questionnaire comprised one additional item on intended behavior to consult again that is not in the scoring, as well as sociodemographic data, overall life satisfaction and an open-ended comment field at the end of the questionnaire.

#### Anxiety and depression

Anxiety and depression were assessed using the French version of the Hospital Anxiety and Depression Scale (HADS), which yields valid and reliable clinical assessments of depression and anxiety [38]. The HADS is a 14-item self-report questionnaire assessing levels of anxiety and depression with 7 items for each subscale [39]. Each item is scored on a 4-point Likert scale. For each subscale, the score is obtained by summing the respective 7 items (subscale scores range from 0 to 21). Each subscale has three severity ranges based on cut-off scores: 0–7 (noncases), 8–10 (mild severity), and 11–21 (moderate or severe severity) [40].

#### Sociodemographic and clinical data

These data included factors that might be related to changes in HRQoL: gender, age, marital status, educational level, occupational status, type of substance dependence, duration of illness, medication introduced, presence of psychiatric and/or somatic comorbidity and origin of the care request. Data related to the physician, including gender, academic qualifications and years of clinical practice, were also noted.

### Statistical analysis

#### Descriptive and comparative analyses

Continuous variables were described by the mean or the median, as appropriate, and categorical variables were described by percentages. Student’s t-test and Pearson’s chi-squared test or Fisher’s exact test were used to compare groups.

#### Bivariate and multivariable analyses

Prior to data analysis, the structure of the 3 questionnaires (SF12 / 2-dimensional, HADS / 2-dimensional and EQS-C / 4-dimensional) was verified in the study sample using a correspondence analysis. Overall, the results obtained from our study sample were satisfactory. For the SF12, two dimensions were found (eigenvalues (cumulative %)) dim 1: 0.50 (81.4%), and dim 2: 0.18 (92.7%). For the HADS, two dimensions were also identified dim 1: 0.43 (45%) and dim 2: 0.22(59.9%). And for the EQS-C questionnaire, 4 dimensions were identified with dim 1: 0.47 (37.7%), dim 2: 0.30 (55%), dim 3: 0.22 (63.9%) and dim 4: 0.12 (67.8%).

Linear regression models were performed to determine the variables associated with changes in PCS and MCS scores (∆HRQoL = HRQoL at 3 months - HRQoL at baseline). Sociodemographic and clinical factors, the influence of anxiety or depression and the early satisfaction with care score were investigated. Factors with *p*-values< 0.2 in the unadjusted analysis were candidates for inclusion in the multivariable models. No selection procedure was applied in the multivariable analysis. The correlations between the variables retained in the models were also tested. Assumptions (e.g., normality, linearity) were verified before making comparisons between groups and building regression models. Analyses were performed using SAS 9.4 (SAS Inst., Cary, NC, USA).

## Results

### Patient characteristics

**Fig. 1:**
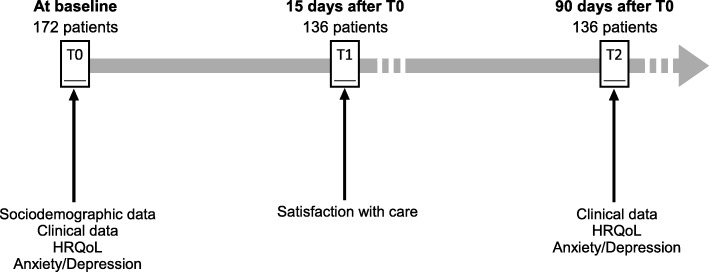
Time points of collection of SUBUSQOL data

A total of 136 patients assessed their satisfaction with care (79.1%), out of 172 patients included in the cohort at baseline (Fig. 1). The sociodemographic and clinical characteristics of the patients are presented separately in Table 1 according to whether the EQS-C was answered. Most of the EQS-C responders were male (82.4%), with a mean age of 39.1 years (SD = 10.5). More than one-third of the patients were married (38.9%), more than half were unemployed (60.3%), and a minority reported having a high school or university level education (16.3%). According to the DSM-IV criteria, 52 (38.2%) patients suffered from alcohol dependence, and 84 patients (61.8%) suffered from opioid dependence. The mean duration of substance dependence was 14.9 years (SD = 11.1). Almost one-third of the patients (30.6%) presented a comorbid Axis I diagnosis. Eleven patients required care while undergoing legally mandated addiction treatment. All of the physicians were currently working with patients with substance dependence, and 100 outpatients were screened by a junior physician (73.5%). In 43.4% of all cases, the patient and physician were of the same gender. After 3 months, the average number of medical sessions completed was 7.3 (SD = 4.5), and a positive change in substance use behavior was observed for 84 (63.2%) outpatients. Patients with opioid dependence (*p* = 0.01) and those who did not have the same gender as their physicians (*p* = 0.04) were significantly more likely to be EQS-C nonresponders; otherwise, the EQS-C nonresponders did not differ from the EQS-C responders in terms of their sociodemographic or clinical characteristics.

**Table 1:** Participant characteristics at baseline (T0)

Characteristics	Satisfaction Outpatient responders *N* = 136		Satisfaction Outpatient nonresponders *N* = 36		*P*-value
	n	Mean (SD) or %	n	Mean (SD) or %	
Age (years)	134	39.1 (10.5)	35	38.1 (10.9)	0.6
Gender					
Male	112	82.4	30	83.3	0.89
Female	24	17.6	6	16.7	
Marital status					
Never married	51	38.9	19	52.8	0.13
Married/live with a partner	51	38.9	14	38.9	
Separated/divorced/widowed	29	22.1	3	8.3	
Educational level					
Primary school	16	11.9	3	8.3	0.63
Secondary school	97	71.9	25	69.4	
High school/university	22	16.3	8	22.2	
Living arrangements					
Alone	44	32.6	12	33.3	0.94
Alone with partner and/or children	59	43.7	16	44.4	
With family or friends	26	19.3	6	16.7	
Homeless	6	4.4	2	5.6	
Occupational status					
Unemployed/student	82	60.3	20	57.1	0.98
Full-time work	41	30.1	11	31.4	
Part-time work	7	5.1	2	5.7	
Retired	6	4.4	2	5.7	
Type of dependence					
Alcohol dependence	52	38.2	6	16.7	0.01
Opioid dependence	84	61.8	30	83.3	
Duration of addiction (years)	136	14.9 (11.1)	36	12.3 (8.8)	0.2
Comorbid axis I diagnosis (yes)	55	30.6	11	40.4	0.28
Origin of the care request					
Patient	100	73.5	25	69.5	0.3
Justice	11	8.1	4	11.1	
Medical care	25	18.4	7	19.4	
Patient-physician gender match (yes)	59	43.4	9	25.0	0.04
Medication initiated during the 3-months follow-up (yes)	53	39.6	9	25.7	0.13
Change in substance use behavior at 3 months (yes)	84	63.2	18	50	0.15
Number of medical sessions during the 3-month follow-up	127	7.3 (4.5)	33	7.5 (5.0)	0.21

### SF-12 and HADS scores at baseline and at the 3-month follow-up

The mean and median PCS, MCS and HADS scores at baseline and at the 3-month period of care are shown in Table 2.

**Table 2:** Self-reported Health status scores at baseline (T0) and a 3-month follow-up (T2) for EQS-C responders

Self-reported Health status	Satisfaction Outpatient responders *N* = 136	
	n	Mean (SD)/ Med*
SF-12 PCS		
PCS at baseline	131	45.2 (9.0) / 45.8*
PCS at 3 months	130	48.0 (7.9) / 50.6*
SF-12 MCS		
MCS at baseline	131	36.1 (10.7) / 34.5*
MCS at 3 months	130	43.0 (10.2) / 42.9*
HADS		
Anxiety subscale score at baseline	128	10.3 (4.5) / 10*
Anxiety subscale score at 3 months	128	8.0 (4.1) / 8*
Depression subscale score at baseline	129	7.9 (4.1) / 8*
Depression subscale score at 3 months	127	5.6 (3.8)/ 5*

*Abbreviations*: *SD* standard deviation, Med* median, *SF-12* Short-Form 12, *PCS* Physical Component Summary, *MCS* Mental health Component Summary, *HADS* Hospital Anxiety and Depression Scale

At T0, the mean SF-12 scores were 45.2 (SD = 9.0) and 36.1 (SD = 10.7) for the PCS and MCS domains, respectively. The mean HADS score for the depression subscale was 7.9 (SD = 4.1), and the mean HADS score for the anxiety subscale was 10.3 (SD = 4.5). The results did not reveal a significant difference between the EQS-C responders and nonresponders in terms of their HRQoL and HADS scores at baseline. After 3 months, the outpatients who responded to the EQS-C showed a positive change in HRQoL scores. The mean SF-12 MCS and SF-12 PCS changes showed significant positive improvements of 7.2 (SD = 12) (*p* < 0.0001) and 2.8 (SD = 8.9) (*p* = 0.0004) points, respectively. The results also showed a positive change in anxiety and depression levels during the first 3 months, with decreases of 2.6 (SD = 3.8) (*p* < 0.0001) and 2.5 (SD = 3.8) (*p* < 0.0001) points, respectively.

### Satisfaction scores

The satisfaction scores are shown in Table 3. The mean overall satisfaction score was 80.8 (SD = 15.2). The mean satisfaction scores were 80.6 (SD = 19.1) for “contact/appointments”, 79.4 (SD = 16.1) for “reception facilities”, 76.5 (SD = 22.6) for “waiting time”, and 82.5 (SD = 16.5) for “consultation with the doctor”.

**Table 3:** Outpatient early satisfaction with care (T1)

Satisfaction with care	EQS-C responders *N* = 136	
	n	Mean (SD) or %
EQS-C scores		
Contact/appointments	132	80.6 (19.1)
Reception facilities	135	79.4 (16.1)
Waiting time	135	76.5 (22.6)
Consultation with the doctor	135	82.5 (16.5)
Overall satisfaction	136	80.8 (15.2)
“I think I will continue attending this department”	136	
Agree		31.6
Fully agree		55.7
Comments on the open-ended EQS-C question “What part of our service do you think could be improved?”	136	
No comment		48.0
Positive comment		27.2
Negative comment		16.1
Mixed comment		8.7

*Abbreviations***:** *EQS-C* Quality of Care Scale in outpatient consultation, *SD* standard deviation

Of the 136 EQS-C responders, 119 (87.3%) intended to consult with the doctor again after the initial consultation. A total of 71 (51.7%) patients made several comments in the open-ended comment field of the EQS-C. Less than a quarter of these comments were negative, and those pertained to waiting and reception.

### Factors associated with HRQoL changes

The results of the bivariate and multivariable analyses are reported in Table 4. Three variables were associated with significant SF-12 PCS improvement from T0 to T2, with a proportion of variance explained (i.e., R^2^) of 0.47. Never being married (β = 5.5; *p =* 0.001), being married (β = 4.0; *p* = 0.02) and having a lower SF-12 PCS score at baseline (β = − 0.6; *p* < 0.0001) were associated with a statistically significant increase in the physical domain score at 3 months. Being legally compelled to undergo addiction treatment (β = − 5.9; *p* = 0.02) was associated with a lower PCS score change compared to personal choice. Two variables were associated with significant SF-12 MCS improvement from T0 to T2, with a proportion of explained variance (i.e., R^2^) of 0.40. Significant increases in the mental health domain were observed at 3 months among outpatients with higher early satisfaction with care (β = 0.1; *p =* 0.02*)* and with lower SF-12 MCS score at baseline (β = − 0.7*; p <* 0.0001).

**Table 4:** Predictors of Health-related Quality of Life change from baseline to the 3-month follow-up

Patients characteristics	∆* SF-12 PCS *N* = 118				∆* SF-12 MCS *N* = 123			
	Bivariate analysis		Multivariate analysis R^2^ = 0.47		Bivariate analysis		Multivariate analysis R^2^ = 0.40	
	*β/Mean*	*P*-value	*β/Mean*	*P*-value	*β/Mean*	*P*-value	*β/Mean*	*P*-value
Age (years)	−0.02	0.84			0.08	0.47		
Gender		0.70				0.89		
Male	3.0				7.3			
Female	2.2				6.9			
Educational level		0.15				0.69		
Primary school	7.2				4.6			
Secondary school	2.1				7.5			
High school/university	3.3				8.0			
Marital status		0.01		0.005		0.47		
Never married	5.6		5.5	0.001	5.6			
Married/live with a partner	2.8		4.0	0.02	8.3			
Separated/divorced/widowed	−1.1		0.0		8.6			
Occupational status		0.4				0.78		
Unemployed	2.3				7.5			
Employed	3.7				6.8			
Type of dependence		0.17				0.38		
Alcohol dependence	1.4				8.4			
Opioid dependence	3.7				6.5			
Duration of addiction (years)	0.04	0.61			−0.09	0.37		
Origin of the care request		0.03		0.014		0.84		
Patient	3.3		0.0		7.0			
Health practitioner	3.7		2.1	0.2	8.5			
Justice	−4.9		−5.9	0.02	6.4			
Comorbid axis I diagnosis		1.0				0.09		0.26
Yes	2.8				8.7		0.0	
No	2.8				4.9		2.0	
Medication initiated during the 3-month follow-up		0.81				0.10		
Yes	3.0				4.9			
No	2.9				8.6			
Change in substance use behavior at 3 months		0.79				0.98		
Yes	2.7				7.2			
No	3.2				7.1			
Self-reported health status at baseline								
HADS depression subscale		0.05		0.77		0.02		0.14
HADS depression subscale score < 8	0.9		0.0		4.6		0.0	
HADS depression subscale score ≥ 8	4.0		−0.4		9.7		−3.0	
HADS anxiety subscale		0.14				0.65		
HADS anxiety subscale score < 8	1.0				6.8			
HADS anxiety subscale score ≥ 8	3.5				7.9			
SF-12 PCS	−0.62	< 0.0001	−0.6	< 0.0001				
SF-12 MCS					−0.67	< 0.0001	−0.7	< 0.0001
EQS-C overall satisfaction score	0.0	0.95			0.17	0.01	0.1	0.02

*Abbreviations***:** *SF-12* Short-Form 12, *PCS* Physical Component Summary, *MCS* Mental health Component Summary, *HADS* Hospital Anxiety and Depression Scale, *EQS-C* Quality of Care Scale in outpatient consultation; *∆ HRQoL = 3-month HRQoL-baseline HRQoL

## Discussion

This study showed low HRQoL at baseline and positive changes in both the physical and mental health domains of HRQoL at the 3-month follow-up among outpatients with substance dependence who were seeking treatment. These results were consistent with previous studies that showed that QoL was low among individuals with SUDs [41–43] and that significant improvements in both the mental and physical dimensions of QoL were found at the three-month follow-up [44–47]. Interestingly, the positive change in the physical domain of HRQoL was smaller than the change in the mental domain. Previous authors have suggested that the lack of improvement within the physical health domain might be due to a shortage of time for allowing substantial improvements or to the intractability of some somatic health problems [48].

The study enabled us to identify several factors linked to a favorable short-term change in HRQoL. First, the improvement in both psychological and physical HRQoL was more pronounced when the score for each self-reported QoL domain was low. This was somewhat expected as it has previously been shown for patients with SUDs [49, 50]. In line with previous studies suggesting that the relationships between patient satisfaction and HRQoL were more significant for the mental health domain [51], the most expected finding was that improvement in the mental component of HRQoL was related to early outpatient satisfaction with care. The current study showed that the mean overall satisfaction score was aligned with studies reporting the mean overall satisfaction scores with French outpatients in medical and surgical departments at public teaching hospitals in Paris [37]. Researchers exploring the relationship between satisfaction with care and QoL among patients with serious mental illness have largely reported a positive relationship between the two [52, 53]. Nevertheless, studies investigating the causal nature of this relationship have remained sparse, and thus far, only a few have found a positive association between satisfaction with care and QoL among those patients [28, 54]. Thus, our results might be even more useful for interventions among clinicians to make them more aware of patients who are unsatisfied early in their care. Moreover, if other studies were to confirm our results, then measures targeted at clinicians might be created to improve early satisfaction and increase the QoL change. As half of the items on the outpatient satisfaction questionnaire used are related to the dimension “consultation with the doctor”, further research is needed to address the influence of the patient-therapist or therapeutic alliance, the therapist’s empathy and the patient-therapist consensus regarding the QoL change, as has been suggested for populations with SUD [55].

Our findings showed that marital status and the origin of the care request were associated with physical HRQoL changes. In our study, never being married appeared to be much more strongly associated with greater improvement in physical HRQoL than being married. This finding was consistent with a previous study on QoL by age group that found greater HRQoL improvement among single participants than among married participants [56]. Being legally compelled to undergo addiction treatment was associated with lower physical HRQoL changes in our sample. Studies investigating coerced drug treatment remain quite limited. However, most of them did not detect significant positive effects of coerced treatment on drug use [57, 58]. Moreover, some of those studies suggested that coerced treatment could alter self-reported health status [59, 60]. We could assume that the coercive nature of mandated addiction treatment could offset its early clinical benefits for HRQoL.

Given the low HRQoL among outpatients with substance dependence who seek care, one would intuitively expect an association between changes in substance use behavior and HRQoL improvement. However, the literature has reported mixed findings and has highlighted that improved QoL may not rely upon abstinence or a reduction in substance use [9]. Although previous studies among patients with SUDs have shown that female gender [9] and an absence of psychiatric comorbidities [16, 49] were associated with better QoL improvement, our study did not find significant associations between gender or comorbidities and HRQoL changes. The same applies for SUD-specific characteristics, such as duration of addiction or change in substance use behavior. The fact that sociodemographics and SUD-specific characteristics did not predict HRQoL changes does not prove that they are unrelated. The small number of patients included in this study reduced the power; therefore, true relationships between gender, age, comorbidities, and duration of dependence and QoL change might not have been detected.

Our study showed that the baseline HRQoL scores of alcohol- and/or opioid-dependent outpatients were much more impaired than those of the general French population [61] and were lower than those of patients with serious mental disorders [62, 63]. Moreover, the scores in the mental domain were more altered than the scores in the physical domain. These findings are consistent with other studies using the SF-12 or the SF-36 questionnaires to measure HRQoL in patients with SUD [49, 64–66]. The mean age of our patient sample was 38.9 years, and one-fifth of the patients were women, which is consistent with the demographics found in addiction research [67, 68]. Moreover, the mean duration of substance dependence was consistent with the time taken to establish substance dependence [69]. In line with a previous study [66] and compared with the proportion of French people over 18 years of age who were unemployed and living alone (9 and 30%, respectively) [70], the high proportion of patients who were unemployed and living alone shows social and familial causes and consequences of substance dependence. Finally, given the prevalence of somatic and mental disorders among substance users and to target patients’ needs and improve care engagement, care skills should be provided through several links with primary health care and mental health system [71].

The study has some limitations. The sample cannot be considered as a reflection of all patients with alcohol or opiate dependence seen in routine medical practice because the participants were recruited through specialty treatment services and their satisfaction was assessed at an early stage of care. Less than a quarter of the participants did not complete the outpatient satisfaction questionnaire, which may limit the generalizability of the results. Nonetheless, very few differences in demographic or clinical characteristics and self-reported health status were found between those who completed the outpatient satisfaction with care questionnaire and those who did not.

To our knowledge, this study is the first to assess the effects of early satisfaction with care on early changes in HRQoL among outpatients starting care for alcohol or opioid dependence. Moreover, the EQS-C response rate of 79,1% demonstrated the patients’ willingness to evaluate their own care, a finding that supports patients’ interest in measuring their satisfaction with ambulatory care in further studies. Considering the response rate of 51.7% for the open-ended comment field of the EQS-C, future qualitative interviews might lead to more information regarding how outpatients with substance dependence perceive satisfaction and could determine which factors might affect early satisfaction with care.

Considering the improvement of HRQoL associated with better early satisfaction with care, this issue will be further explored among both patients and clinicians in future studies and in clinical practice to suggest improvements in the early care of these patients. The present study also has several methodological merits. First, patient satisfaction was measured shortly after inclusion. Thus, the level of early satisfaction with care that the patients expressed was independent from later improvements in their HRQoL. Our study design also required patients to complete the satisfaction questionnaire at home, thus avoiding a variant of the Hawthorne effect [72]. Moreover, it is important to note that the multivariable models showed an explained variance of 40 and 47%.

## Conclusion

This study confirms the poor HRQoL of outpatients with substance dependence who were starting care at our French specialized addiction treatment centers and their major improvement after 3 months. The longitudinal design enabled us to identify early satisfaction with care as a factor linked to mental HRQoL change at the 3-month follow up. These findings have several implications. Early satisfaction with care among outpatients with substance dependence should be improved because better early satisfaction with care has been related to better HRQoL improvement. The first step should be to assess satisfaction and HRQoL regularly during outpatient follow-up. From a theoretical perspective, the determinants of patients’ satisfaction with early care must be better identified to identify areas of improvement. This could help clinicians better target their patients’ needs, an action that has been recognized to enhance treatment engagement, care adherence and therapeutic success. Moreover, these findings, if communicated to patients, could enhance their motivation to enter outpatient treatment.

## Data Availability

Data will not be shared to protect the participants’ anonymity.

## Abbreviations

DSM IV: Diagnostic and Statistical Manual of Mental Disorders, fourth edition
EQS-C: quality of care satisfaction questionnaire in the outpatients’ consultation
HADS: Hospital Anxiety and Depression Scale
HRQoL: Health-related quality of life
QoL: Quality of life
SF-12: Short-Form 12 questionnaire
SUBUSQOL: SUBstance Users Satisfaction and Quality Of Life
SUD: Substance use disorders
